# Study of the growth mechanism of a self-assembled and ordered multi-dimensional heterojunction at atomic resolution

**DOI:** 10.1007/s12200-023-00091-2

**Published:** 2023-11-16

**Authors:** Zunyu Liu, Chaoyu Zhao, Shuangfeng Jia, Weiwei Meng, Pei Li, Shuwen Yan, Yongfa Cheng, Jinshui Miao, Lei Zhang, Yihua Gao, Jianbo Wang, Luying Li

**Affiliations:** 1 Wuhan National Laboratory for Optoelectronics, Huazhong University of Science and Technology, Wuhan, 430074 China; 2 Ministry-of-Education Key Laboratory for the Green Preparation and Application of Functional Materials, Hubei Collaborative Innovation Center for Advanced Organic Chemical Materials, School of Materials Science and Engineering, Hubei University, Wuhan, 430061 China; 3 Center for Electron Microscopy, MOE Key Laboratory of Artificial Micro- and Nano-Structures and the Institute for Advanced Studies, School of Physics and Technology, Wuhan University, Wuhan, 430072 China; 4 State Key Laboratory of Infrared Physics, Shanghai Institute of Technical Physics, Chinese Academy of Sciences, Shanghai, 200083 China

**Keywords:** Multi-dimensional composite materials, Ordered heterostructures, Self-assembly, Growth mechanism

## Abstract

Multi-dimensional heterojunction materials have attracted much attention due to their intriguing properties, such as high efficiency, wide band gap regulation, low dimensional limitation, versatility and scalability. To further improve the performance of materials, researchers have combined materials with various dimensions using a wide variety of techniques. However, research on growth mechanism of such composite materials is still lacking. In this paper, the growth mechanism of multi-dimensional heterojunction composite material is studied using quasi-two-dimensional (quasi-2D) antimonene and quasi-one-dimensional (quasi-1D) antimony sulfide as examples. These are synthesized by a simple thermal injection method. It is observed that the consequent nanorods are oriented along six-fold symmetric directions on the nanoplate, forming ordered quasi-1D/quasi-2D heterostructures. Comprehensive transmission electron microscopy (TEM) characterizations confirm the chemical information and reveal orientational relationship between Sb_2_S_3_ nanorods and the Sb nanoplate as substrate. Further density functional theory calculations indicate that interfacial binding energy is the primary deciding factor for the self-assembly of ordered structures. These details may fill the gaps in the research on multi-dimensional composite materials with ordered structures, and promote their future versatile applications.

**Graphical Abstract:**

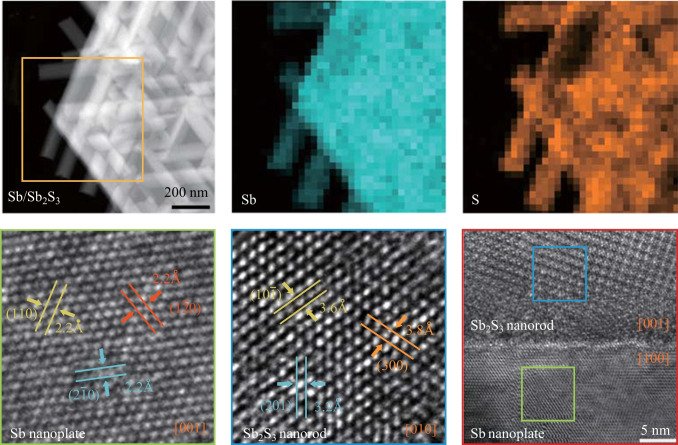

**Supplementary Information:**

The online version contains supplementary material available at 10.1007/s12200-023-00091-2.

## Introduction

The application fields of multi-dimensional heterojunctions are rapidly expanding due to the excellent properties of materials in different dimensions [1–4]. In order to achieve further improved performances, a practical and common method is to combine materials with different dimensions and physical properties [5–7]. However, the reported combining methods are either based on disordered self-assembly or include very complicated manufacturing processes. In this paper, ordered self-assembly of quasi-one-dimensional (quasi-1D) Sb_2_S_3_ nanorods on quasi-two-dimensional (quasi-2D) Sb nanoplates is realized by a simple solution method, which can contribute to development of the fabrication of ordered composite materials with multiple dimensions.

The novel 2D single-element semiconductor material Antimonene (an allotrope of Sb) was first reported in 2015 based on density functional theory (DFT) calculations [8]. According to extensive theoretical and experimental research in recent years, 2D antimonene show excellent performances, such as high specific capacity, high carrier mobility, good stability, and adjustable band gap. It shows great application prospects in the fields of energy, optoelectronics, medicine, and so on [9–14]. Antimony sulfide has also been extensively studied by researchers because of its high specific capacity, high absorption coefficient of visible light, adjustable band gap, and abundant availability, and also has a wide range of applications in energy, optoelectronics, catalysis, and other fields [15–18].

At present, the methods for preparing multi-dimensional materials mainly include physical mixing and chemical mixing. Physical mixing is comparatively simple and economical [19]. For example, Ji et al. reports the feasibility of obtaining high gravimetric capacities and theoretical energy densities, beyond five times those of conventional Li-ion systems, via physical mixing of conductive mesoporous carbon framework and sulphur nanofillers [20]. However, physical mixing does less well than chemical mixing in terms of microscopic mixing uniformity. Embedding a small percentage of well-dispersed graphene nanosheets in epoxy coatings can lead to remarkably improved anticorrosion performance and wear resistance. However, this composite method normally results in disordered assembly, and there is space for further improvement in uniformity [7]. In 2020, Ge et al. reported successful growth of polyaniline core arrays on nitrogen-doped graphene nanosheets in an orderly manner using the *in-situ* dilution polymerization method, and the product shows excellent electrochemical performance [5]. However, the complex processing is not suitable for mass production. Thus, the challenge to simplify the ordered assembly process is a significant issue in order to realize extensive applications of this growth method.

In this paper, a simple hot injection solution method is utilized to achieve ordered self-assembly of quasi-1D antimony sulfide nanorods on a quasi-2D antimonene nanoplate with six-fold symmetry. The thermal injection method is a technique for pyrolyzing precursors to prepare materials. Injecting the precursor solution into the high temperature reactor is the primary idea behind this procedure. When compared to other preparation methods, the hot injection method can quickly produce high-quality items. This approach is flexible, because it can use a variety of solvents and reactants and is efficient for mass production. To propel the future applications of multi-dimensional heterostructures, and to realize ordered self-assembly of homogenous 2D materials and those of other dimensions as a general method, it is necessary to clarify the morphological and structural characteristics of the composite system, as well as the underlying growth mechanism.

Here, the quasi-1D antimony sulfide nanorods spontaneously arranging in six-fold symmetry on quasi-2D antimonene nanoplate are systematically characterized by comprehensive transmission electron microscopy (TEM) techniques, and their formation mechanism is studied by first-principles calculations. This work fills the research gap on ordered self-assembly of multi-dimensional composite materials, and provides new insights into nano-devices based on the ordered heterostructures of multi-dimensional materials.

## Results and discussion

**Fig. 1:**
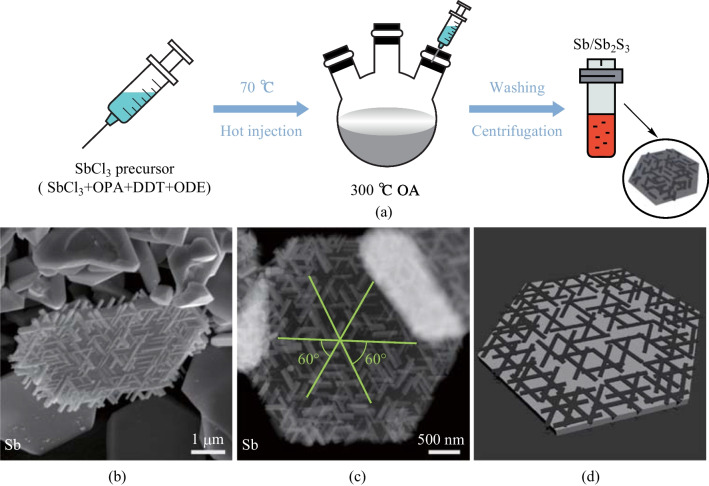
Synthesis and morphological characterization of the multi-dimensional composites. **a** Sketch of the sample preparation process. **b** SEM and **c** HAADF images of the composite material. **d** Schematic model of the composite material

The preparation process of quasi-1D antimony sulfide nanorods on quasi-2D antimonene nanoplates is shown in Fig. 1a. During the hot injection process, the growth of antimonene nanoplates is accompanied by the reduction of antimony chloride in an alkaline solution. After that, the generated antimony sulfide nanorods are attached to the surface of the antimonene nanoplates and arranged in six-fold symmetry during the cooling process. (Refer to the detailed experimental procedure in the experimental section (Sect. 4).) The final products include a large number of quasi-2D antimonene nanoplates and a composite of quasi-1D antimony sulfide and quasi-2D antimonene, which are stored in ambient air. The products are dispersed in toluene solvent and drop-coated on a copper grid as the sample for TEM observation.

The scanning electron microscopy (SEM) images of the sample in Fig. 1b and Fig. S1 show that in the obtained samples, there are mainly four distinct morphologies: first, pure nanoplates with lateral dimensions of around 4 μm, which are hexagonal in shape; second, nanoplates that are not expanded symmetrically with trapezoidal shape; third, single crystals with average lateral size of about 800 nm; fourth, composites of nanorods and nanoplates (the nanoplates involved are also around 4 μm in size), which are the main research object in the current work. Figure S2 shows the high- and low-magnification TEM and SEM images of the composite heterojunctions to help more clearly identify the heterojunctional structure. According to Fig. 1b and c, the nanorods are about 500 nm in length and 60 nm in width, their size range can be well matched with the fabrication of quasi-2D electronic devices, and also has good compatibility with the requirements of catalysis and energy applications [21]. The ordered assembly of nanorods on quasi-2D nanoplate could be a major advance in the synthesis of spontaneously ordered composite heterostructures.

While the SEM image (Fig. 1b) shows the ordered arrangement of nanorods on a trapezoidal-shaped nanoplate, the high-angle annular-dark-field image (HAADF) of multi-dimensional composite heterostructures with a specific hexagonal nanoplate as substrate is presented in Fig. 1c and Fig. S2. The overlapping regions of pairs of nanorods lying along different directions appear brighter, indicating a comparatively larger projected thickness with contributions from both nanorods of the pair in local regions. The spatial arrangement of nanorods on the nanoplate is not perfectly uniform, which shows a higher density of nanorods in the marginal areas as compared to the central regions. Concerning the angular arrangement, it is obvious that all the nanorods are arranged in six-fold symmetrical directions, which are at 60 ℃ with respect to each other. In other words, without the influence of any external factors, the nanorods would spontaneously arrange along six-fold symmetrical directions on the nanoplates, as schematically shown in Fig. 1d.

**Fig. 2:**
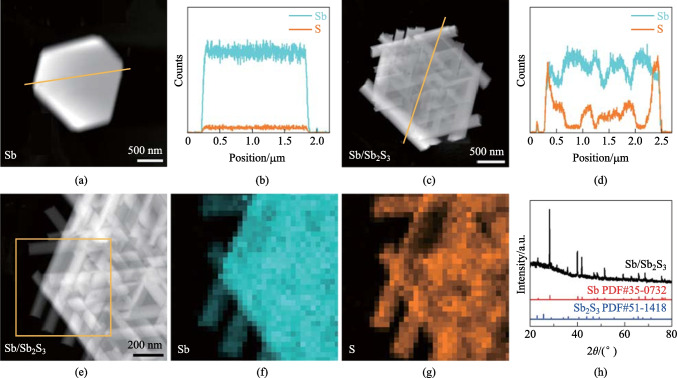
Chemical analysis of the composite heterostructures. **a**, **c** HAADF images of pure nanoplate and a composite of nanorods/nanoplate, respectively. **b**, **d** Corresponding elemental line profiles of the regions labeled by yellow lines in **a** and **c**, respectively. **e**–**g** HAADF image and 2D elemental maps of the composite of nanorods/nanoplate. **h** XRD spectra of the obtained product

In order to determine the composition of the obtained multi-dimensional composite material, electron energy-dispersive spectroscopy (EDS) elemental line profiles and maps of transmission electron microscope and X-ray diffraction (XRD) spectra are collected for analysis. EDS line profiles are obtained on a pure nanoplate and a composite of nanorods/nanoplates, respectively. Figure 2a and b show the HAADF image of a pure nanoplate and the corresponding elemental line profiles. When the background is carefully removed, the line profiles show obvious Sb and weak S signals across the nanoplate. The latter could be attributed to the sulfhydryl groups derived from mercaptan attached to the surface of nanoplates. For comparison, Fig. 2c and d show the HAADF image of a composite of nanorods/nanoplates and the corresponding elemental line profiles. Similarly, both Sb and S signals appear, but the content of S is much higher for the composite than it is for pure nanoplate, and more S signals distribute on the marginal areas of the nanoplate, which is in accordance with the observed denser distribution of nanorods there.

EDS elemental maps are also obtained from the composite nanorods/nanoplates. Figure 2e–g show the HAADF image and 2D elemental maps of the composite material. It is observed that the Sb signals of the nanorods stretching out of the nanoplate are much weaker than those of the nanoplate region, as the latter have contributions from both nanorods and nanoplate. The S signals, on the other hand, are distributed solely in regions that include nanorods. In addition, quantitative compositional analysis is performed (Fig. S3) on both the overlapping regions of nanorod and nanoplate (1) and pure nanorod (2). The results show that the atomic ratio of S:Sb is 1:0.53 for pure nanorod, and in the overlapping area of nanorod and nanoplate, the S:Sb ratio is 1:7.32. Although the atomic ratio obtained from EDS spectra is not quantitatively accurate without calibration based on reference samples, the variational trend emphasizes the much greater contribution from the nanoplate for the Sb signal.

Moreover, the XRD spectrum (Fig. 2h) of the composite of nanorods/nanoplates can be attributed to both Sb and Sb_2_S_3_. While the peak intensities related to Sb are much higher, the weak peak intensities of Sb_2_S_3_ could be due to the much smaller size and projected thickness of nanorods as compared to nanoplates. While the quasi-2D antimonene nanoplates are synthesized by simple thermal injection method, the quasi-1D antimony sulfide nanorods are generated via the reaction of sulfur ions in the solution with antimony ions on the antimonene nanoplate surfaces. Considering the limited amount of sulfur ions in the solution, and the resultant finite size of antimony sulfide nanorods as compared to antimonene nanoplates, it is reasonable to expect substantially weaker peaks for Sb_2_S_3_ in the XRD spectrum. In addition, there are some additional peaks that belong neither to Sb nor to Sb_2_S_3_, for example the peaks at ~ 32°. The most plausible scenario is that Sb possesses structures of other crystal system, namely monoclinic structure of Sb in addition to the common hexagonal crystal system (PDF#26-0101), with its (200) plane corresponding to 32.291° in the XRD spectrum. However, because of the large thicknesses of such Sb nanocrystals, it is difficult to verify the above assumption via TEM characterizations. Thus, it can be preliminarily determined that the composite material is composed of quasi-2D antimonene as substrate and spontaneously ordered quasi-1D antimony sulfide. It is worth noting that the size of the nanorods is related to the dosage of the injected precursors and thiols. Therefore, it is possible to tailor the relative size of the composite heterostructure for future device applications.

**Fig. 3:**
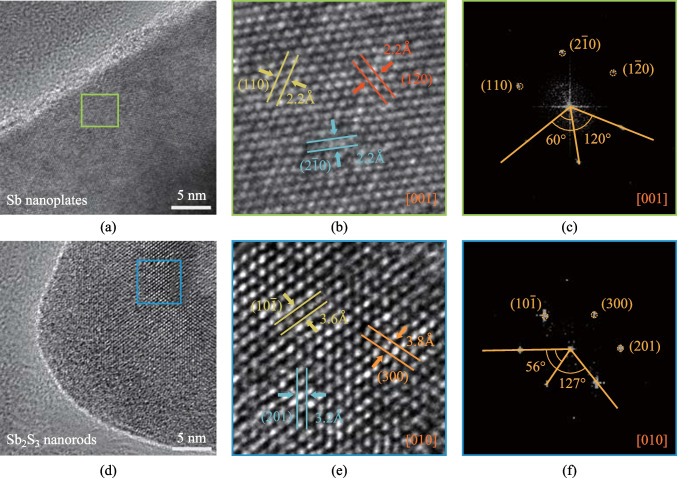
Plan-view structural characterization of the Sb_2_S_3_ nanorod/Sb nanoplate. **a**, **d** Plan-view HRTEM images of selective nanoplate and nanorod regions in the same composite material, respectively. **b**, **e** Enlarged images of regions frames by a green box and a blue box in **a** and **d**, respectively. **c**, **f** FFTs of **b** and **e**, respectively

In order to understand the formation mechanism of this composite heterostructure from a structural perspective, the crystal structure of the composite material is analyzed from both plan-view and cross-sectional directions, so as to gain a comprehensive understanding of the spontaneous joining of Sb_2_S_3_ nanorods and Sb nanoplate in an spontaneously ordered manner. In Fig. 3, specific regions of nanoplate and nanorod from a composite of nanorods/nanoplates are selected for analysis to determine their relative orientation. Figure 3a is a high-resolution transmission electron microscopy image (HRTEM) of the Sb nanoplate. The enlarged image of the green box region in Fig. 3a shows a six-fold symmetric structure with [001] projection (Fig. 3b), and the marked lattice spacings are all 0.22 nm, which can be labeled as (110), ($2\overline{1}0$), and ($1\overline{2}0$) of hexagonal Sb, respectively. Figure 3c is the corresponding fast Fourier transform (FFT) of Fig. 3b, and the included angles between the three marked planes are 60° and 120°.

Figure 3d is an HRTEM image of the nanorod region of the composite material. Likewise, it is observed that the marked lattice spacings are 0.36, 0.38, and 0.32 nm, which correspond to ($10\overline{1 }$), (300), and (201) planes of orthorhombic Sb_2_S_3_ projected along [010] axis. The angles of the three crystal planes marked in Fig. 3e and f are 56° and 127°, respectively. Figure S4 is the atomic-resolution HAADF image of the nanorod region: the bright spots perfectly coincide with the Sb columns of Sb_2_S_3_ projected along [001] direction, as can be seen from the overlapping atomic model of Sb_2_S_3_, which is also projected along [001] axis. The S columns are missing from the HAADF image due to the much smaller atomic number of S (*Z*_S_ = 16) as compared to Sb (*Z*_Sb_ = 51). The above results further confirm that the composite of nanorods/nanoplates is spontaneously ordered quasi-1D Sb_2_S_3_ nanorods on quasi-2D Sb nanoplate with [010] Sb_2_S_3_ parallel to [001] Sb.

**Fig. 4:**
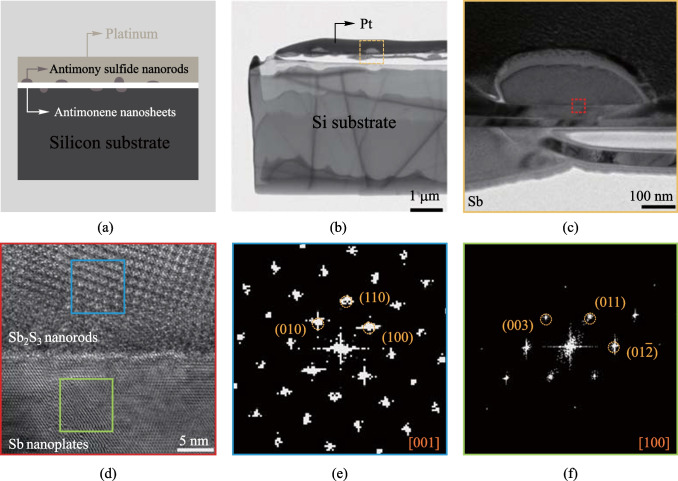
Cross-sectional structural characterization of the Sb_2_S_3_ nanorod/Sb nanoplate composite. **a** Sketch of the cross-sectional view of Sb_2_S_3_ nanorod/Sb nanoplate heterostructure for FIB sample preparation. **b** TEM image of the cross-sectional sample. **c** Magnified TEM image of the area framed in the yellow dotted box in **b**. **d** HRTEM image of the area framed in the red box in **c**. **e** and **f** FFTs of the selected areas framed in the upper blue box and lower green box in **d**, respectively

Besides plan-view characterization, the cross-sectional view of the Sb_2_S_3_ nanorod/Sb nanoplate composite can reveal comprehensive information about the orientational relationship of the multi-dimensional heterostructure. The composite material was suspended on a silicon substrate and covered by a platinum layer for protection during sample preparation, as shown in Fig. 4a. Using the focused ion beam (FIB) technique, a thin slice was cut out perpendicularly to the growth direction of the nanorod including the nanorod/nanoplate heterostructural interface (Fig. 4b), and the yellow dotted box region is magnified and presented in Fig. 4c, which includes one Sb_2_S_3_ nanorod and the underneath Sb nanoplate.

Figure 4d is an atomic-scale HRTEM image at the nanorod/nanoplate interface. While the upper Sb_2_S_3_ and lower Sb regions show entirely different lattice fringes, the abrupt structural change at the interface can lead to a large strain field, which is reflected by the seemingly thin amorphous layer at the interface. It is very common to have lattice distortion at the heterostructure (Fig. S5) due to different lattice parameters and structures of the contacting materials, which would lead to different local orientations as compared to the surrounding areas. Thus, the absence of periodic lattice information in the interfacial region might not be attributed to an amorphous layer but to a high level of strain field. Moreover, because the sample is prepared by FIB, the different sputtering rates of the two materials might also lead to varying thicknesses especially at the interface region, and different focusing conditions. It is well known that the high-resolution STEM-HAADF technique is very sensitive to focusing, which could also lead to local blurring. In the right part of Fig. S5, it could be seen that the continuous extending of lattice planes from lower Sb layer to upper Sb_2_S_3_ layer with no obvious sign of an amorphous layer.

The FFTs of the blue and green box regions in Fig. 4d are presented in Fig. 4e and f, respectively, which indicates that the [001] axis of the Sb_2_S_3_ nanorod is parallel to the [100] axis of the Sb nanoplate. Based on the orientational relationship analysis from both plan view and cross-sectional view, the 3D schematic diagram of the crystal plane orientations of the composite heterostructure can be clearly determined with [010] Sb_2_S_3_//[001]Sb and [001]Sb_2_S_3_//[100]Sb.

**Fig. 5:**
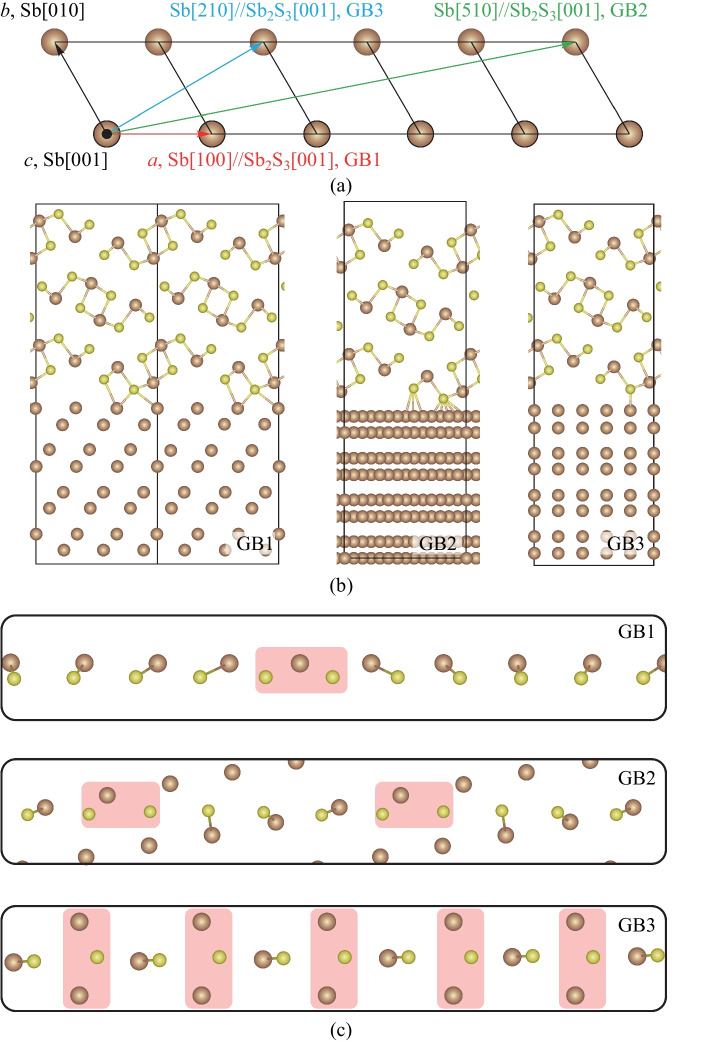
Theoretical analysis of the composite heterostructure. **a** Orientational relationships of three types of GBs between Sb_2_S_3_ (010) and Sb (001). **b** Atomic models for GB1, GB2 and GB3. Brown and yellow balls represent Sb and S, respectively **c** Bonding nature of relaxed Sb_2_S_3_-Sb interface. The shallow regions show longer Sb–S bonds, and only two atomic layers are shown for simplicity

In order to further explore the underlying growth mechanism of ordered Sb_2_S_3_ nanorods on Sb nanoplates from a theoretical point of view, the density functional theory (DFT) was used to calculate the binding energies between Sb_2_S_3_ and Sb with different crystal plane orientations. The grain boundary (GB) models of the composite material were based on fully relaxed Sb (*R-3m*, *a* = 4.38 Å, *c* = 11.59 Å) and Sb_2_S_3_ (*Pbnm*, *a* = 11.09 Å, *b* = 11.47 Å, *c* = 3.86 Å). Three types of GB were constructed by matching Sb (001) and Sb_2_S_3_ (010) interfaces along different orientations, as shown in Fig. 5a and Table 1. Figure 5b shows the crystal structures of GBs with a total of 408, 262, and 100 atoms for GB1, GB2, and GB3, respectively. The outmost two layers of Sb and one layer of Sb_2_S_3_ could relax while the remains were fixed to mimic the bulk region. Periodic boundary conditions were adopted to avoid long-range interface dipole influence. The DFT calculations were performed using the VASP code [22, 23] with the standard frozen-core projector augmented-wave (PAW) [24, 25] method. The cutoff energy for basis functions was 350 eV. The generalized gradient approximation (GGA) of the Perdew-Burke-Ernzerh (PBE) functional was used for exchange–correlation [26]. All atoms were relaxed until the Hellmann–Feynman forces on them are below 0.03 eV/Å. For structural relaxations, the k-point meshes were chosen such that the product of the number of k points and the corresponding lattice parameter was at least 40 Å. The zero-damping DFT-D3 method of Grimme was used for Van der Waals interactions in Sb_2_S_3_ and corresponding GBs [27].

**Table 1:** Lattice mismatches along two orthogonal vectors (*u* and *v*) and total energies for three GBs

GB type	Interface parameter				GB energy per square meter/(J⋅m^−2^)
	*u*	Lattice mismatch/%	*v*	Lattice mismatch/%	
GB1	Sb[100]//Sb_2_S_3_[001]	0.75	Sb[210]//Sb_2_S_3_[100]	2.51	0.312
GB2	Sb[510]//Sb_2_S_3_[001]	3.96	Sb[-130]//Sb_2_S_3_[100]	4.40	0.362
GB3	Sb[210]//Sb_2_S_3_[001]	1.79	Sb[010]//Sb_2_S_3_[100]	1.37	0.360

The smallest lattice mismatches of GBs as shown in Table 1 indicate a minor in-plane stress, which is highly desirable for heterojunction modeling. The main difference between the three GBs lies in the relative atomic arrangement between S and Sb at the interface. The compact and strong binding between Sb–S at the interface can effectively stabilize the GB structure. From Fig. 5c, we can see that GB1 has the strongest Sb–S binding at the interface (shorter Sb–S bond lengths and more compact packing between Sb and S) due to the parallel arrangement between Sb ([100]Sb) and S ([001]Sb_2_S_3_), where most S atoms are located on top of Sb atoms. When rotating Sb_2_S_3_ by 10.9° with respect to GB1, the distance between Sb and S is enlarged, resulting in a loose binding of GB2. A similar situation can be found in GB3, where a 30° rotation is applied, more Sb–S bonds hold longer bond lengths when the S atom is located on the bridge site of the neighboring two Sb atoms. The calculated GB energies in Table 1 show that GB1 has the lowest energy, conforming to the strongest Sb–S binding at the interface of GB1.

**Fig. 6:**
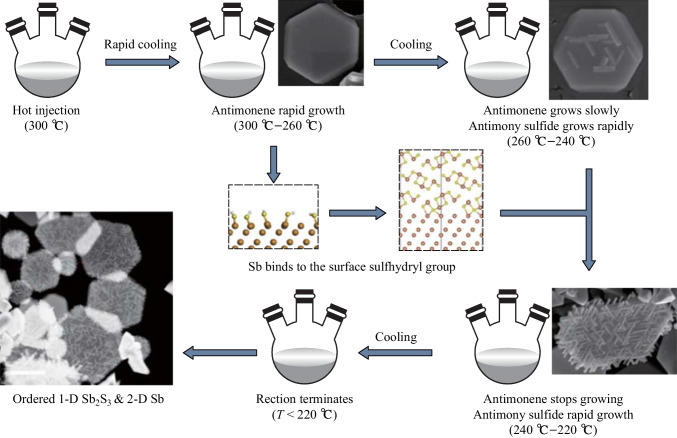
Schematic diagram showing formation process of the Sb_2_S_3_ nanorod/Sb nanoplate composite material

Based on the above experiments and analyses, it can be speculated that the formation process of the composite material is as shown in Fig. 6. Surface dangling bonds must be purposefully added to the surface of two-dimensional materials in order to make them spontaneously form composites with other materials. The observations in this work were, that after the initial hot injection, the solvent was cooled rapidly, and 2D antimonene nanoplates started to form at 300 ℃ down to 260 ℃. As the temperature continued to decrease, the number of atomic layers of antimonene increased, and the 2D material gradually transformed into quasi-2D material. At this stage, due to the continuous decrease of temperature and increase of thickness, sulfhydryl dangling bonds appeared on the surface of antimonene. Since the neighboring Sb atoms did not have enough energy to form Sb–Sb bonds, Sb tends to replace H and form Sb–S bonds. (The bond energy of Sb–Sb is 1217 kJ/mol, and that of S–H is 339 kJ/mol.) Therefore, antimony sulfide begins to grow on the surface of the antimonene nanoplate.

Because the grain boundary energy of the Sb_2_S_3_/Sb hetero-interface is the lowest at GB1, as presented in Table 1, and the antimonene (001) surface has six-fold symmetry, antimony sulfide nanorods spontaneously order themselves in directions that allow the lowest overall grain boundary energy of the Sb_2_S_3_/Sb heterostructure. When the temperature drops to 220 ℃, the reaction stops, and the spontaneously ordered multi-dimensional composite material is finally stabilized.

## Conclusions

Ordered self-assembly of quasi-one-dimensional antimony sulfide nanorods on a quasi-two-dimensional antimonene nanoplate was successfully realized by an economical thermal injection method. Comprehensive transmission electron microscopy characterizations reveal that the composite of Sb_2_S_3_ nanorods and Sb nanoplates has good crystallinity. Quasi-1D antimony sulfide nanorods are spontaneously ordered into six-fold symmetry on the quasi-2D nanoplate, and their orientational relationship is revealed by plan-view and cross-sectional structural characterizations: [010]Sb_2_S_3_//[001]Sb and [001]Sb_2_S_3_//[100]Sb.

Furthermore, this work explores, theoretically, the formation mechanism of the composite material. Because the crystal structure of antimonene [001] has six-fold symmetry, the binding energy of antimonene and antimony sulfide is also distributed with six-fold symmetry. In the [100], [010], and [$\overline{11 }0$] directions of antimonene, the binding energy with antimony sulfide is the lowest, which would lead to the spontaneous ordering of antimony sulfide nanorods on the antimonene nanoplate. The most important factor for this ordered self-assembled growth is the binding energy of the contact crystal planes of the two materials, and the lowest binding energy is preferred for self-assembly growth mode. This growth mechanism reveals that other hetero-junctional materials might also be designed and manufactured based on the symmetry-related binding energy distributions. The interfacial binding energy controlled spontaneous ordering of materials in different dimensions can greatly simplify the synthesis process of multi-dimensional materials with ordered structure, and pave the way for fabricating two-dimensional devices and circuits with potential applications.

## Experimental section

Synthesis of composite material: The first step was to prepare SbCl_3_-DDT. SbCl_3_ (0.912 g, 4 mmol), dodecanethiol (DDT, 4 mL), and octadecene (ODE, 6 mL) were added into a 50 mL 3-neck flask, degassed in vacuum for 2 h at 110 ℃ and purged with nitrogen, which was then heated in the nitrogen atmosphere at 150 ℃ until all SbCl_3_ reacted with DDT. Since SbCl_3_-DDT solution precipitates out of ODE at room temperature, it was necessary to preheat it to 60 ℃ before use. The second step was the synthesis of quasi-1D antimony sulfide nanorods and quasi-2D antimonene nanoplate composites, Oleylamine (0.5 mL) and ODE (4.0 mL) were added in a 50 mL three-necked flask. The reaction mixture was degassed in vacuum for 30 min at 110 ℃ and purged with nitrogen. Next, the flask was heated to 300 ℃, and then 1.0 mL of SbCl_3_-DDT solution was injected into the reaction system swiftly. After that, the reaction was stopped immediately, and antimonene nanoplates were obtained by centrifugation three times (8000 r/min, 5 min).

Preparation of Cross-Sectional TEM Samples: the composite material dispersed in toluene was sonicated for 10 min. Some were loaded onto copper grids with holey carbon films for TEM observations, while others were transferred to Si substrates for preparation of cross-sectional specimens by focused ion beam (FIB) using Tescan Gaia 3. During the preparation, Pt was deposited on the top surface beforehand to act as a protection layer. Then the nanoflakes were milled by Ga ions with an energy of 30 keV until the selected regions became electron-transparent. Finally, Ga ions with relatively lower beam energies (5 and 2 keV) were employed to remove damage layers covering surfaces of the cross-sectional samples.

Structural characterizations: The morphology of the Sb_2_S_3_ nanorods/Sb nanoplates composite was characterized by SEM (Nova NanoSEM450). HRTEM and HAADF-STEM images of the Plan-view and the Cross-sectional structural characterization of the composites were obtained using JEM-ARM200CF with double Cs correctors operated at 200 kV. XRD patterns were measured with an X-ray diffractometer (Bruker, D2 Phaser with Cu-Kα (*λ* = 1.5405 Å) radiation) at room temperature in the 2*θ* range of 20°–80°.

### Supplementary Information

Below is the link to the electronic supplementary material.

Supplementary file1 (PDF 467 KB)

## Data Availability

The data that support the findings of this study are available from the corresponding author, upon reasonable request.
