# Supplementary material for: Study of the growth mechanism of a self-assembled and ordered multi-dimensional heterojunction at atomic resolution
Source: Front Optoelectron. 2023 Nov 16;16(1):35. doi: 10.1007/s12200-023-00091-2 (PMC10654331; doi:10.1007/s12200-023-00091-2)
Supplement: Supplementary file 1 — Supplementary file1 (PDF 467 KB) [file 12200_2023_91_MOESM1_ESM.pdf]

Supporting Information

Study of the growth mechanism of self-assembled and ordered multi-dimensional heterojunction at atomic resolution

Zunyu Liu[1], Chaoyu Zhao[2], Shuangfeng Jia[3], Weiwei Meng[3], Pei Li[3], Shuwen Yan[1], Yongfa Cheng[1], Lei Zhang*[,2], Yihua Gao[1], Jianbo Wang[3], Luying Li*[,1]

[1]Wuhan National Laboratory for Optoelectronics, Huazhong University of Science and Technology, 1037 Luoyu Road, Wuhan, Hubei, 430074, China

[2]Ministry-of-Education Key Laboratory for the Green Preparation and Application of Functional Materials, Hubei Collaborative Innovation Center for Advanced Organic Chemical Materials, School of Materials Science and Engineering, Hubei University, No. 368, Friendship Avenue, Wuhan, Hubei, 430061, China

[3]Center for Electron Microscopy, MOE Key Laboratory of Artificial Micro- and Nano-Structures and the Institute for Advanced Studies, School of Physics and Technology, Wuhan University, Wuhan, Hubei, 430072, China

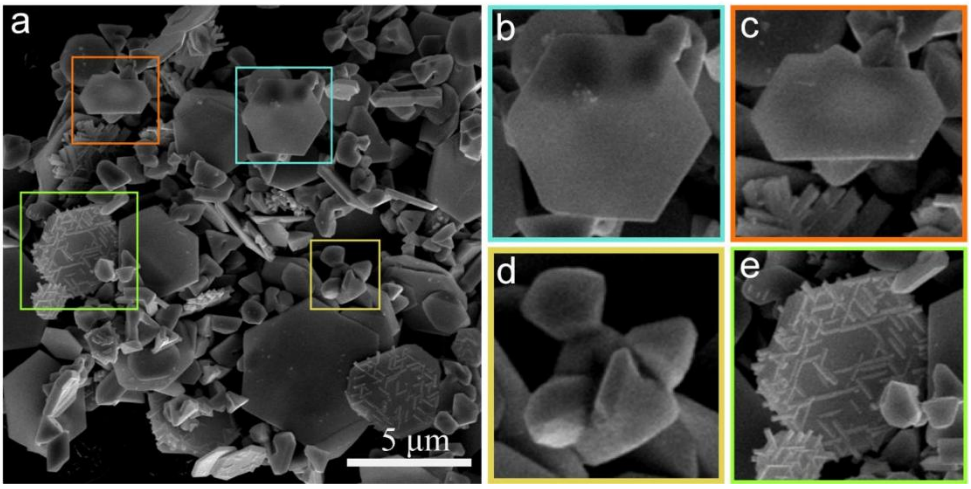

Figure S1. a) SEM image showing four distinct morphologies of the sample. b) Pure nanoplate of hexagonal shape. c) Nanoplate of trapezoidal shape. d) Single crystals of various shapes. e) Composite of nanorods and nanoplates.

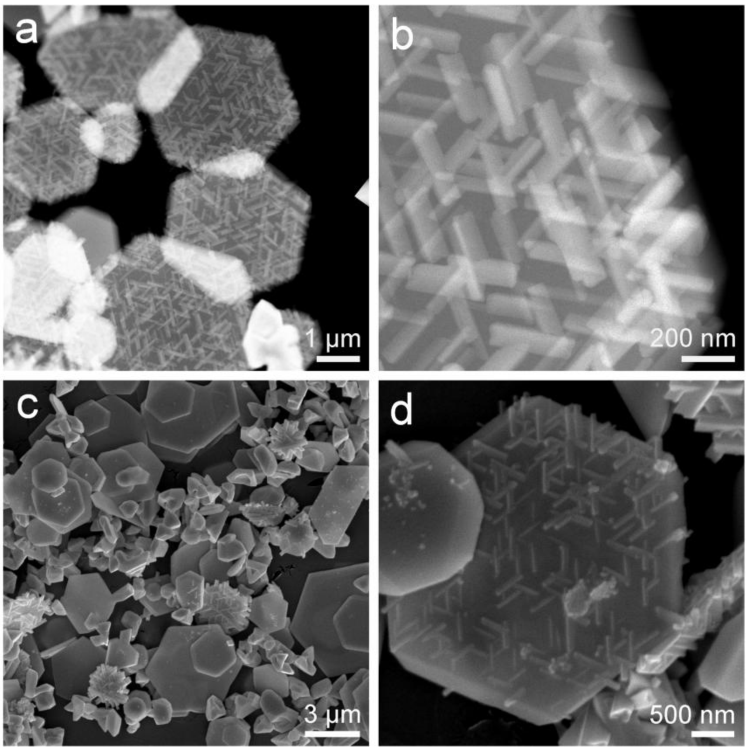

Figure S2. TEM (a-b) and SEM (c-d) images of the composite heterojunctional materials at different magnifications.

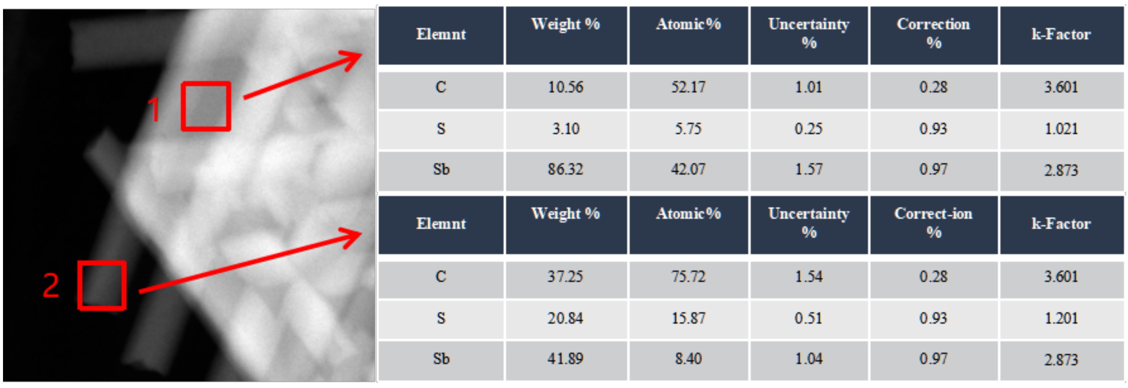

Elemnt	Weight %	Atomic%	Uncertainty %	Correction %	k-Factor
C	10.56	52.17	1.01	0.28	3.601
S	3.10	5.75	0.25	0.93	1.021
Sb	86.32	42.07	1.57	0.97	2.873
Elemnt	Weight %	Atomic%	Uncertainty %	Correct-ion %	k-Factor
C	37.25	75.72	1.54	0.28	3.601
S	20.84	15.87	0.51	0.93	1.201
Sb	41.89	8.40	1.04	0.97	2.873

Figure S3. The HAADF image of the composite material and the corresponding quantitative chemical analysis using EDS. Region 1 and region 2 correspond to nanoplate/nanorod composite and pure nanorod, respectively.

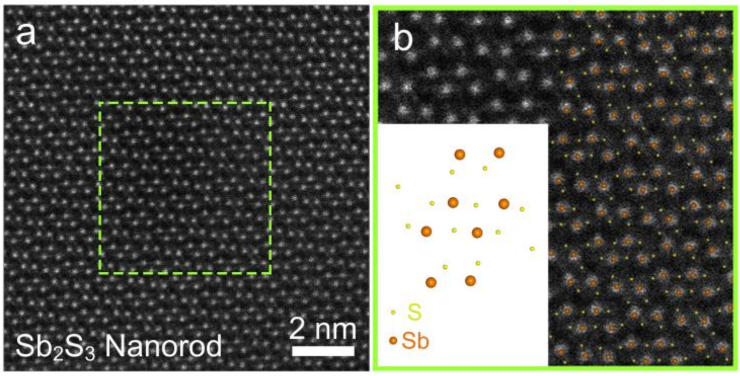

Figure S4. a) The atomic resolution HAADF image of pure nanorod region in the composite materials. b) Magnified image of the red dotted box region in (a), which is projected along [001] axis. The bottom left inset is the atomic model of Sb23 with [001] projection. The atomic model is overlapped on top of the right-hand side of the HAADF image, where the bright spots coincide with the Sb atomic columns.

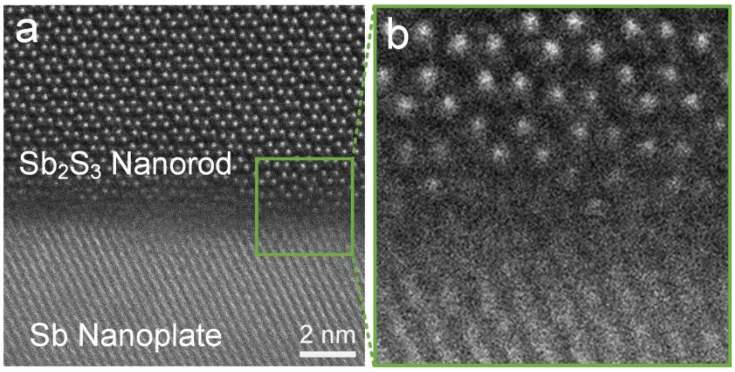

Figure S5. The atomic resolution HAADF image of Sb_2S_3 nanorod/ Sb nanoplate hetero-interface. While Sb_2S_3 is perfectly projected along its [001] axis, Sb is slight tilted off its [010] axis.
